# Level of utilization and provider-related barriers to the use of hydroxyurea in the treatment of sickle cell disease patients in Jos, North-Central Nigeria

**DOI:** 10.4314/ahs.v21i2.36

**Published:** 2021-06

**Authors:** Akinyemi OD Ofakunrin, Edache S Okpe, Tolulope O Afolaranmi, Rasaq R Olaosebikan, Patience U Kanhu, Kehinde Adekola, Nantok Dami, Atiene S Sagay

**Affiliations:** 1 Department of Paediatrics, University of Jos / Jos University Teaching Hospital, Jos, Nigeria; 2 Department of Community Medicine, University of Jos / Jos University Teaching Hospital, Jos, Nigeria; 3 Department of Pharmacology and Experimental Therapeutics, Thomas Jefferson University, Philadelphia Pennsylvania, USA; 4 Division of Hematology-Oncology, Northwestern University, Feinberg School of Medicine, USA; 5 Department of Obstetrics and Gynaecology, University of Jos / Jos University Teaching Hospital, Jos, Nigeria

**Keywords:** Hydroxyurea utilization, barriers to hydroxyurea, sickle cell disease, Nigeria

## Abstract

**Background:**

Hydroxyurea is underutilized by sickle cell health-care providers in Nigeria despite available evidence of its effectiveness in reducing the manifestations and complications of sickle cell disease (SCD).

**Objectives:**

To assess the level of utilization and provider-related barriers to the use of hydroxyurea in SCD therapy in Jos, Nigeria.

**Methods:**

A cross-sectional study conducted among 132 medical doctors providing care for SCD patients. Data on sociodemographics, utilization and barriers to hydroxyurea use were obtained. The barriers were fed cumulatively into the logistic regression model as predictors of utilization.

**Results:**

Of the 132 care providers, 88 (67%) had been in medical practice for ≥6years. The level of utilization of hydroxyurea was 24.2%. The significant barriers that predicted the non-utilization of hydroxyurea included lack of expertise (OR=5.1; 95% CI=2.65–9.05), lack of clinical guidelines (OR=3.84; 95% CI=2.37–14.33), fear of side-effects (OR=0.50; 95% CI=0.22–0.68) and doubt about its effectiveness (OR=0.30; 95% CI=0.20–0.90).

**Conclusion:**

The level of utilization of hydroxyurea in the treatment of SCD among the care providers is sub-optimal with the lack of expertise in its use identified as the most prominent barrier. There is an urgent need for the training of sickle cell care-providers and the development of clinical guidelines on hydroxyurea use.

## Introduction

Sickle-cell disease (SCD) is an inherited blood disorder that is characterised by ongoing haemolytic anaemia and recurrent vaso-occlusive events which result in episodic painful crises, organ dysfunctions, poor quality of life and when untreated, early mortality. 1–3 An estimated 25 million people are affected globally with the majority of them living in sub-Saharan Africa where Nigeria accounts for the highest burden of the disease. 4–5

Despite the huge burden of SCD in Nigeria, its treatment has been mainly symptomatic with focus on pain relief, malaria prophylaxis and folic acid supplementation.^6^ However, a disease-modifying therapy for SCD namely hydroxyurea has been discovered and it is widely available. 7

Hydroxyurea is a ribonucleotide reductase inhibitor that has been demonstrated by several clinical studies as a safe and efficacious medication capable of reducing the manifestations and complications of SCD in both adults and children.^8^–^11^ Globally, it has emerged as a major advancement in the treatment of SCD as it is one of the currently approved medications with the capability of modifying the disease process.^7^ The National Institute of Health (NIH) in 2014 and the British Society of Haematology in 2018 both recommended that all patients with the disorder and their family members should be educated about hydroxyurea therapy and all affected children from age nine months and above should be offered hydroxyurea regardless of clinical severity to reduce SCD-related complications and end organ failure.^12^, ^13^

In Nigeria, available reports suggest that the medication is underutilized by the sickle cell health-care providers while the barriers to its utilization in the treatment of SCD among the providers are yet to be elucidated. 6,14–16 This study, therefore, assessed the level of utilization and barriers to the use of hydroxyurea in the treatment of SCD among the health care-providers in Jos, North-Central Nigeria.

## Materials and methods

### Study area

The study was conducted in the four tertiary hospitals in Jos, Plateau State, North-Central Nigeria. Based on the 2006 population census, Plateau State had an estimated population of 3.2 million people; and the population of Jos was 736,016.^17^ The four tertiary health institutions served as referral centres for the provision of advanced care for SCD patients. The hospitals receive referrals from various health facilities in Plateau State and seven neighbouring States. These hospitals are the Jos University Teaching Hospital (JUTH), Plateau State specialist hospital (PSSH), Our Lady of Apostles Hospital (OLAH), and Bingham University Teaching Hospital (BhUTH). Based on the hospital records, over 2500 patients with SCD are being catered for at these facilities.

### Study population

The study was conducted among medical doctors providing medical care for SCD patients in the four hospitals. In the studied setting, SCD patients are cared for by the Paediatricians, Haematologists, Family Physicians and General Practitioners.

### Study design

This was a cross-sectional study to assess the level of utilization of hydroxyurea and the barriers to its use in the treatment of SCD patients. The study was conducted between September and November 2018.

### Inclusion criteria

All doctors involved in the medical care of sickle cell disease patients in the four hospitals who consented to participate in the study.

### Exclusion criteria

Medical doctors who refused to participate in the survey and those who have not attended to any SCD patient in the last twelve months were excluded.

### Sample size determination

The sample size for this study was determined using the appropriate sample size estimation formula for a cross-sectional study.^18^ The parameters used included the standard normal deviate at 95% confidence interval (1.96), q is the complementary probability (1 – p), d is the precision of the study set at 0.05 and p is the proportion of doctors with adequate utilization of hydroxyurea in the treatment of SCD patients in a previous similar study which was 9% (0.09).^19^ This gave a minimum sample size of 132 after 5% of the calculated sample size was added to cater for non responses, poor or incomplete responses.

### Sampling method

A list of all the medical doctors providing medical treatment for SCD patients in all the four hospitals was made giving a total of 242 doctors of which 112 doctors were in JUTH, 57 in PSSH, 22 in OLAH and 51 in BhUTH. Thereafter, a proportion to size technique was used to determine the number of doctors that will be sampled in each of the four hospitals. This was done by dividing the total number of doctors providing medical services to SCD patients in each of the hospitals by the total number of doctors providing medical services to SCD patients in all the hospitals multiplied by the sample size. Thereafter, a list of all the eligible doctors in all the hospitals was made and serialized. A computed generated table of random numbers was used to select 61, 28, 31 and 12 doctors from the serialised list of the doctors in JUTH, BhUTH, PSSH and OLAH respectively. Furthermore, the doctors whose number corresponded to the one selected by the computer were approached and sampled until the sample size was met. Data was collected from each participant using a pretested, structured and self-administered questionnaire. The data collected included the socio-demographics, utilization and barriers to the utilization/prescription of hydroxyurea.

### Data analysis

#### Scoring and grading of responses

In this study, hydroxyurea was considered to have been ‘utilized’, if a provider has prescribed hydroxyurea to any SCD patient in the last 12 months and ‘not utilized’, if otherwise. The level of utilization of hydroxyurea was assessed by determining the number of providers who had prescribed hydroxyurea to their eligible SCD patients in the last 12 months, and this was also corroborated by determining the proportion of their eligible patients who are on hydroxyurea. Patients' eligibility was based on the 2014 National Institute of Health (NIH) expert panels' recommendation and 2018 British Society of Haematology guideline for children and adults with sickle cell disease. 12, 13 When the proportion of eligible patients on hydroxyurea was greater than 10%, it was categorised as ‘adequate utilization’ and when it was equal to or less than 10%, it was regarded as ‘inadequate utilization’. 19 Ten percent cut off was adopted for the adequacy of utilization in this study because the level of uptake of hydroxyurea in Nigeria is still low with some studies reporting between 0 and 2.6% of eligible patients being on hydroxyurea. 14–15

The barriers to the utilization of hydroxyurea which was assessed as explanatory variables included: statement of affirmation of fear of side effects, expertise in the use of hydroxyurea (this involves being conversant with the dosing regimen, treatment monitoring and management of possible side effects), availability of clinical guidelines, cost of medication and monitoring of treatment, doubt about the effectiveness of hydroxyurea, perception of patients refusal etc. In order to reduce recall / information bias, the research questions were carefully selected and the duration for the recall of prescription of hydroxyurea was restricted to a maximum of 12 months.

### Statistical analysis

All the returned questionnaires were reviewed for completeness and thereafter serialized in preparation for data entry. Data analysis was carried out using SPSS version 23.0 for Windows (SPSS, Chicago, IL). Descriptive statistical analysis was carried out on qualitative variables such as sex, speciality, cadre etc of the respondents. These and other explanatory variables such as the provider-related barriers were presented in frequency tables and expressed in frequencies and percentages. The barriers were fed cumulatively into the logistic regression model as predictors of utilization of hydroxyurea. Chi-square test was used to test the association between some demographic characteristics of the respondents and utilization of hydroxyurea. Adjusted odds ratio and 95% confidence interval were used as point and interval estimates respectively with a P-value of <0.05 considered statistically significant.

### Ethical considerations

Ethical approval (JUTH/DCS/ADM/127/XVIII/1156) was obtained from the Health Research Ethicsal Committee of the Jos University Teaching Hospital. Permission was sought and gotten from the various heads of the participating hospitals and departments. Written informed consent was sought and obtained from every respondent before enrolment into the study. Assurance of anonymity and confidentiality of all information supplied was also given.

## Results

### Socio-Demographic Characteristics

A total of 132 medical doctors who had been providing medical care for sickle cell disease patients participated in this study. Seventy-four (56.1%) of the respondents were males while 58 (43.9%) were females. Sixty-one (46.2%) of the respondents were family physicians while paediatricians and haematologists accounted for 13.6% and 8.3% respectively. Eighty-eight (67%) of the studied population had been in medical practice for upward of six years while 80 (60.6%) of them affirmed to have attended to more than ten SCD patients in the last 6 months (Table 1).

**Table 1:** Demographic characteristics of the respondents

Variable	Frequency (n=132)	Percentage (100.0)
**Sex**		
Male	74	56.1
Female	58	43.9
**Speciality**		
Paediatrics	18	13.6
Family medicine	61	46.2
Haematology	11	8.3
General Practice	42	31.8
**Cadre**		
Medical officers	51	38.6
Resident doctors	52	39.4
Consultants	29	22.0
**Years of practice**		
1–5years	44	33.4
6 -10 years	46	34.8
>10 years	42	31.8
**Facility of practice**		
JUTH	61	46.2
BhUTH	28	21.2
PSSH	31	23.5
OLAH	12	9.1
**Number of SCD patients** **seen in the last 6 months**		
1–5	22	16.7
6–10	30	22.7
>10	80	60.6
**Categories of patient seen**		
Children only	18	13.6
Adults only	16	12.1
Both	98	74.3

JUTH- Jos University Teaching Hospital, BUTH – Bingham University Teaching Hospital, Plateau State Specialist Hospital, OLAH – Our Lady of Apostle Hospital

### Utilization of hydroxyurea in the treatment of sickle cell disease patients

Ninety-three (70%) of the respondents had never discussed hydroxyurea as a treatment option with any SCD patient. About two-thirds (65.9%) of the respondents admitted that they lacked or had poor expertise in the use of hydroxyurea for the treatment of sickle cell disease while only 32 (24.2%) of the respondents had prescribed hydroxyurea for the treatment of sickle cell disease in the last 12 months. Only 13 (9.8%) of the providers had >10% of their eligible patients on hydroxyurea (Table 2).

**Table 2:** Utilization of hydroxyurea in the treatment of sickle cell disease patients among all the respondents

Variable	Frequency	Percentages
	n=132	
**Ever discussed hydroxyurea as a treatment** **option with any SCD patient**		
Yes	39	29.5
No	93	70.5
**Expertise in the use of hydroxyurea**		
Good	11	8.3
Fair	34	25.8
Poor/None	87	65.9
**Prescribed hydroxyurea in the last 12 months**		
Yes	32	24.2
No	100	75.8
**Proportion of eligible SCD patients on** **hydroxyurea**		
None	100	75.8
<5%	11	8.3
5 – 10%	8	6.1
11–50%	13	9.8
>50%	0	0.0
**Utilization of hydroxyurea**		
Utilized	32	24.2
Not utilized	100	75.8
**Adequacy of utilization**		
Adequate	13	9.8
Inadequate	119	90.2

### Comparison of the utilization of hydroxyurea by speciality, cadre and years of practice

Table 3 shows the utilization of hydroxyurea based on speciality, cadre and years of practise among the study participants. The proportion of haematologists (81.8%) and paediatricians (72.7%) who had prescribed hydroxyurea within the last 12 months were more than the proportions of family physicians (13.1%) and the general practitioners (4.8%) and the difference was statistically significant (p< 0.0001) (Table 3).

**Table 3:** Comparison of the utilization of hydroxyurea by speciality, cadre, and years of practice

Variable	Utilized n (%)	Not utilized n (%)	Total	χ2	P-value
**Speciality**					
Paediatrics	13(72.2)	5(27.8)	18		
Family medicine	8(13.1)	53(86.9)	61		
Haematology	9(81.8)	2(18.2)	11		
General Practice	2 (4.8)	40(95.2)	42	**55.2**	**<0.0001**
**Total**	**32**	**100**	**132**		
**Cadre**					
Medical officers	2(3.9)	49(96.1)	51		
Resident doctors	22(42.3)	30(57.7)	52		
Consultants	8(27.6)	21(72.4)	29	**20.88**	**<0.0001**
**Total**	**32**	**100**	**132**		
**Years of practice**					
1–5years	3(6.8)	41(93.2)	44		
5 -10 years	18(39.1)	28(60.9)	46		
>10 years	11(26.2)	31(73.8)	42	**12.91**	**0.002**
**Total**	**32**	**100**	**132**		

Among the different cadres, the proportion of resident doctors (42.3%) who utilized hydroxyurea was more than the other cadres (3.9% and 27.6%- medical officers and consultants respectively) and the difference was statistically significant (p<0.0001).

### Provider-related barriers to the utilization of hydroxyurea

Lack of expertise was the most frequent barrier to the use of hydroxyurea for the treatment of SCD as 86 (65.2%) of the respondents expressed this as a barrier. Other main barriers identified among the respondents included lack of clinical guidelines on hydroxyurea use (56.1%), fear of side effects (37.1%), and 22% expressed doubt about the effectiveness of hydroxyurea in achieving desired treatment outcomes as a barrier (Table 4).

**Table 4:** Providers-related barriers to the utilization of hydroxyurea

Variables	Frequency	Percentage
Lack of expertise in hydroxyurea use	86	65.2
Lack of clinical guidelines on hydroxyurea use	74	56.1
Fear of side effects	49	37.1
Doubt about the effectiveness of hydroxyurea	29	22.0
Patient did not meet the criteria for initiating the treatment	25	18.9
Perception of patients refusal	23	17.4
Fear of poor adherence to medication by the patients	16	12.1
Not usually comfortable treating SCD patients	8	6.1
Cost of medication and monitoring of treatment	5	3.8

NB – There were multiple responses

SCD – Sickle cell disease

### Factors influencing the non-utilization of hydroxyurea

The odds of non-utilization of hydroxyurea was 5.1 times higher in providers with no expertise in its use compared to those with some level of expertise (OR =5.1; 95% CI =2.65–9.05; P<0.0001). Similarly, the odds of non-utilization of hydroxyurea was 3.8 times higher among providers who did not have clinical guidelines on hydroxyurea use compared to those who had (OR =3.84; 95% CI =2.37–14.33). Other barriers that predicted the non-utilization of hydroxyurea included fear of side-effects (OR =0.50; 95% CI =0.22–0.68; P=0.019) and doubt about its effectiveness in SCD management (OR =0.30; 95% CI =0.20–0.90; P=0.002). (Table 5).

**Table 5:** Factors influencing the non-utilization of hydroxyurea in the study population

Variables	Odds ratio	95% CI	P-value
Speciality			
Paediatrics	1.570	0.010 – 3.765	0.766
Family medicine	1.634	0.015 – 1.784	0.687
Haematology	0.200	0.100 – 0.900	**0.004**
General Practice	1	-	-
**Cadre**			
Resident doctors	2.20	0.179 – 5.053	0.355
Medical officers	0.90	0.725 – 1.010	0.213
Consultants	1	-	-
**Barriers**			
Fear of side effects			
Absent	0.50	0.215 – 0.684	**0.019**
Present	1	-	
Expertise in the use of hydroxyurea in SCD			
Absent	5.05	2.647 – 9.048	**<0.0001**
Present	1		
Lack of clinical guidelines on hydroxyurea use			
Yes	3.84	2.366 -14.335	**<0.0001**
No	1	-	
Effectiveness of hydroxyurea			
Not doubtful	0.30	0.200 -0.901	**0.002**
Doubtful	1	-	
Perception of patients refusal	0.26	0.194 -1.869	0.200
Fear of poor patients' medication adherence	0.69	0.349 – 4.958	0.451
Not comfortable treating	1.30	0.318 – 9.076	0.759
SCD patients			
Patient did not meet the criteria for initiating therapy	1.07	0.780 – 5.420	0.887
Cost of medication and monitoring of treatment	0.94	0.459 – 4.995	0.919

## Discussion

The level of utilization of hydroxyurea by the providers in this study was low, as only about a quarter of the respondents reported its use within the last 12 months. Besides, only a few of the respondents ‘adequately’ utilized hydroxyurea in the management of their SCD patients. This level of utilization (24.2%) is higher than the level reported in the studies conducted in Zaria, Nigeria in 2007 and 2017 where abysmally low levels of utilization of 0% and 2.6% respectively were reported. 14–15 The level is however lower than those reported in the studies conducted in the United States.^19^–^20^ Our finding implies that most of the eligible patients are being denied the opportunity of receiving this disease-modifying therapy. Consequently, these patients could be at risk of more frequent sub-clinical and clinical vaso-occlusive events and haemolysis which could result in frequent painful crises, hospital admissions, early complications including irreversible organ damages and poor quality of life. 1–3 The reason for the low level of utilization of hydroxyurea in this study may be due to poor knowledge among the respondents as about two-thirds of the interviewed providers self-reported poor or no expertise in the use of hydroxyurea. A previous study had identified poor provider knowledge as a reason for the low level of utilization of therapies among sickle cell disease patients.^21^ Also, in contrast to our study, the design in the US surveys focused mainly on specialist care providers (haematologists) who most probably must have had the requisite training and retraining as well as established protocol on the use of hydroxyurea.^22^ However, in most low-middle-income countries, there is a dearth of specialist care providers and hence the need for training of more health care providers (non-haematologists) to boost the skilled manpower needed to reduce the burden of this disease by filling the gap.^23^, ^24^

Furthermore, some barriers were reported by the respondents to have affected the utilization of hydroxyurea in the treatment of their patients.

This study found that the most common barrier to the utilization of hydroxyurea among the respondents was the lack of expertise in its use in the treatment of sickle cell disease. The lack of expertise involves the aspect of dosing regimen, monitoring and management of possible side effects in the prospective patients. The National Institutes of Health Consensus Development Conference Statement on Hydroxyurea had similarly identified ‘limited number of physicians who have expertise in the use of hydroxyurea for sickle cell disease’ as a barrier.^23^ This finding implies that continuous medical education and capacity development should be regularly provided for doctors who care for sickle cell disease patients to keep them abreast of the developments and tools that could be beneficial to their patients.

Lack of clinical guidelines on the use of hydroxyurea in the treatment of sickle cell disease was also identified as a barrier in this study. This is in synergy with the findings from recent studies which acknowledged the lack of treatment guidelines as a barrier to the use of hydroxyurea, especially in low-resource countries. 25, 26 The development of clinical guidelines could help to standardize practice, boost the confidence and enhance the expertise of the physicians. It is worth noting that there's an on-going effort by the Sickle Cell Disease Stroke Prevention in Nigeria (SPIN) trial towards establishing local clinical guidelines for hydroxyurea use, especially for stroke prevention.^27^ Furthermore, as it has been reported in previous studies, fear of side effects of hydroxyurea was another reason for not prescribing hydroxyurea by this study's participants.^23^, ^25^, ^28^ Some of the side effects reported in order of concern were bone marrow suppression, carcinogenicity and infertility. It has been well documented that the bone marrow suppressive effect of hydroxyurea is dose-dependent and easily reversible with no life-threatening event reported from its occurrence providing adequate monitoring is instituted. 11, 29, 30 Consequently, provision of adequate monitoring by the health care providers requires that they have requisite knowledge and expertise in the use of hydroxyurea for SCD management. Similarly, hydroxyurea at therapeutic dosage has not been reported to be associated with an increased risk of cancer among sickle cell disease patients who are on the medication compared to those not on it or the general population.^31^ Therefore, it will be unjustifiable to withhold beneficial treatment from the patients on account of inference that is not evidence-based. Regarding infertility, sickle cell disease has been associated with infertility especially in males. Also, a few studies have reported the contribution of hydroxyurea treatment to male infertility.^32^ However, the area of reproductive epidemiology in sickle cell disease is vastly understudied and requires further research to obtain concrete evidence.^32^ Nevertheless, the risk of untreated SCD cannot be compared to the risk associated with HU therapy, especially in low-middle-income countries. Therefore, HU use appears to be highly justified. Doubts about the drug's effectiveness were also found to have constituted a significant barrier to its use. This finding is similar to what was reported by a study conducted among haematologists/oncologists in Florida and North Carolina.^33^ Local studies on the effectiveness and safety of hydroxyurea may be needed to remove this barrier and allay the fear of toxicity of this medication in low- middle -income countries.^11^ Finally, the cost of the medication and monitoring of therapy was reported as a barrier by a few of the respondents. This may, nonetheless, be a very important barrier among the patients, especially in low resource countries, and it's worth exploring through a further research.

We are not unmindful that our study, being a cross-sectional survey, may be limited by the possibility of recall bias. Also, the availability of facilities for hydroxyurea treatment monitoring could also constitute a barrier to its prescription but this was not assessed in this study.

## Conclusion

The level of utilization of hydroxyurea by the providers in this study was low. Lack of expertise in the use of hydroxyurea in the treatment of sickle cell disease was the most important barrier preventing the doctors from utilizing/prescribing hydroxyurea to their patients. Other identified significant barriers included lack of clinical guidelines on hydroxyurea use in sickle cell disease, fear of side effects and doubt about the effectiveness of the drug.

The information from this survey has provided some insights into the barriers to the utilization of hydroxyurea in a low-middle income setting. The barriers need to be addressed so that SCD patients could benefit from this life-saving and disease-modifying therapy. There is, therefore, an urgent need to engage the sickle cell care-providers in training on the use of hydroxyurea in the therapy of SCD as well as the development of clinical guidelines. The training could be done within the framework of local practice, thereby providing the local capacity necessary to expand the safe use of hydroxyurea.
